# Crystal structure of [tris­(4,4′-bi­pyridine)]­diium bis­(1,1,3,3-tetra­cyano-2-eth­oxy­propenide) trihydrate

**DOI:** 10.1107/S2056989016012160

**Published:** 2016-08-05

**Authors:** Fatima Setifi, Arto Valkonen, Zouaoui Setifi, Sami Nummelin, Rachid Touzani, Christopher Glidewell

**Affiliations:** a Laboratoire de Chimie, Ingénierie Moléculaire et Nanostructures (LCIMN), Université Ferhat Abbas Sétif 1, Sétif 19000, Algeria; b Department of Chemistry, University of Jyvaskyla, PO Box 35, FI-40014 Jyvaskyla, Finland; c Biohybrid Materials, Department of Biotechnology and Chemical Technology, Aalto University, FI-02150 Espoo, Finland; d Laboratoire de Chimie Appliquée et Environnement, LCAE–URAC18, COSTE, Faculté des Sciences, Université Mohamed Premier, BP 524, 60000, Oujda, Morocco; e Faculté Pluridisciplinaire Nador BP 300, Selouane, 62702, Nador, Morocco; f School of Chemistry, University of St Andrews, Fife KY16 9ST, Scotland

**Keywords:** crystal structure, hydro­thermal synthesis, polynitrile anions, mol­ecular structure, hydrogen bonding

## Abstract

The title hydrated salt, C_30_H_26_N_6_^2+^·2C_9_H_5_N_4_O^−^·3H_2_O, was obtained as an unexpected product from the hydro­thermal reaction between potassium 1,1,3,3-tetra­cyano-2-eth­oxy­propenide, 4,4′-bi­pyridine and iron(II) sulfate hepta­hydrate. The cation lies across a twofold rotation axis in the space group *I*2/*a* with the other components all in general positions. In the cation, the H atom linking the pyridine units is disordered over two adjacent sites having occupancies of 0.66 (4) and 0.36 (4), *i.e.* as N—H⋯N and N⋯H—N. The water mol­ecules of crystallization are each disordered over two sets of atomic sites, having occupancies of 0.522 (6) and 0.478 (6) for one, and 0.34 (3) and 0.16 (3) for the other, and it was not possible to reliably locate the H atoms associated with these partial-occupancy sites. In the crystal, four independent C—H⋯N hydrogen bonds link the ionic components into a three-dimensional network.

## Abstract 2

In the title hydrated salt, which was obtained from the hydro­thermal reaction between between potassium 1,1,3,3-tetra­cyano-2-eth­oxy­propenide and 4,4′-bi­pyridine in the presence of iron(II) sulfate hepta­hydrate, the ionic components are linked into a three-dimensional network by C—H⋯N hydrogen bonds.

## Chemical context   

**Figure 1:**
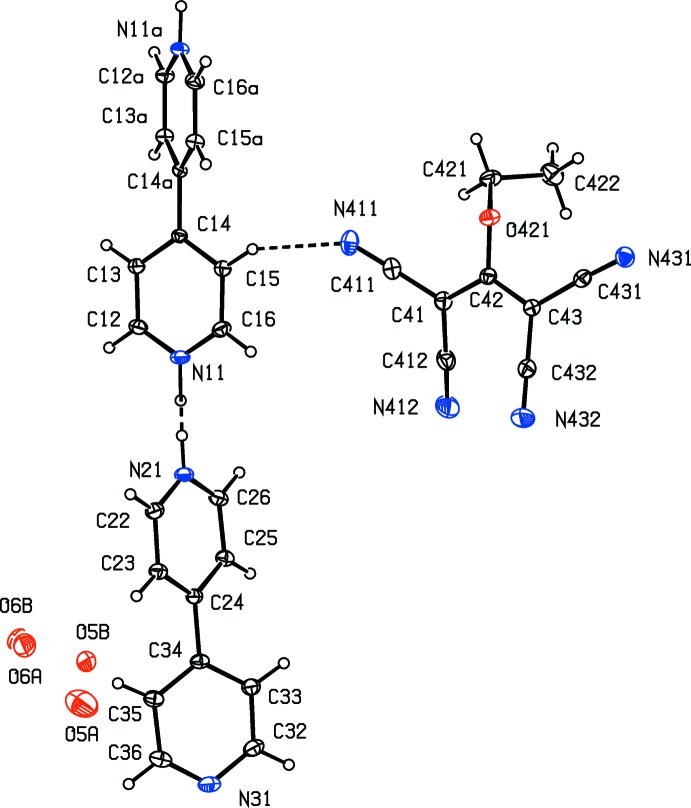
The independent components of the structure of compound (I), showing the atom-labelling scheme, the complete central 4,4′-bipy unit and the hydrogen bond (shown as a dashed line) between the cation and anion within the selected asymmetric unit. Displacement ellipsoids are drawn at the 30% probability level and the atoms marked with ‘a’ are at the symmetry position (−*x* + 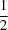, *y*, −*z* + 1). The partially occupied water sites have refined occupancies as follows: O5*A* 0.522 (6), O5*B* 0.478 (6), O6*A* 0.34 (3) and O6*B* 0.16 (3).

In recent years, the use of polynitrile anions as coordinating ligands for the construction of polymeric architectures with inter­esting properties has been a burgeoning subject in materials and coordination chemistry (Thétiot *et al.*, 2003 ▸; Benmansour *et al.*, 2007 ▸; Atmani *et al.*, 2008 ▸). These anions are versatile structural components, leading to many different architectures in zero, one, two or three dimensions, and incorporating most of the 3*d* transition metals (Benmansour *et al.*, 2008 ▸, 2010 ▸, 2012 ▸; Yuste *et al.*, 2009 ▸; Setifi, Domasevitch *et al.*, 2013 ▸; Setifi, Setifi *et al.*, 2013 ▸; Setifi, Lehchili *et al.*, 2014 ▸). This versatility is based on two main properties of these ligands: (i) the ability to act as bridges, given the linear and rigid geometry of the cyano groups, and (ii) the possibility of functionalization with different potentially coordinating groups that leads to a high variety of coordination modes. To take advantage of this behaviour we have been using these organic anions in combination with other chelating or bridging neutral co-ligands to explore their structural and electronic characteristics of the resulting complexes, particularly with reference to mol­ecular materials exhibiting inter­esting magnetic exchange coupling behaviour. During the course of attempts to prepare such complexes with 4,4′-bi­pyridine, we isolated the title compound (I) (Fig. 1 ▸ and Scheme 1), whose structure is reported here.

## Structural commentary   

The structure of compound (I) consists of a [tris­(4,4′-bi­pyridine)]diium dication, [(4,4′-bipy)-H-(4,4′-bipy)-H-(4,4′-bipy)]^2+^, two 1,1,3,3-tetra­cyano-2-eth­oxy­propenide anions, [(NC)_2_CC(OEt)C(CN)_2_]^−^, and three water mol­ecules. The cation lies across a twofold rotation axis, selected for the reference cation as that along (0.25, *y*, 0.5), while the other components all lie in general positions. Within the cation, the H atom linking the 4,4′-bipy units is disordered over two adjacent sites having occupancies of 0.66 (4) and 0.36 (4), and the two independent water mol­ecules are also disordered, both over two atomic sites, with one having occupancies of 0.522 (6) and 0.478 (6) and the other having occupancies of 0.34 (3) and 0.16 (3).
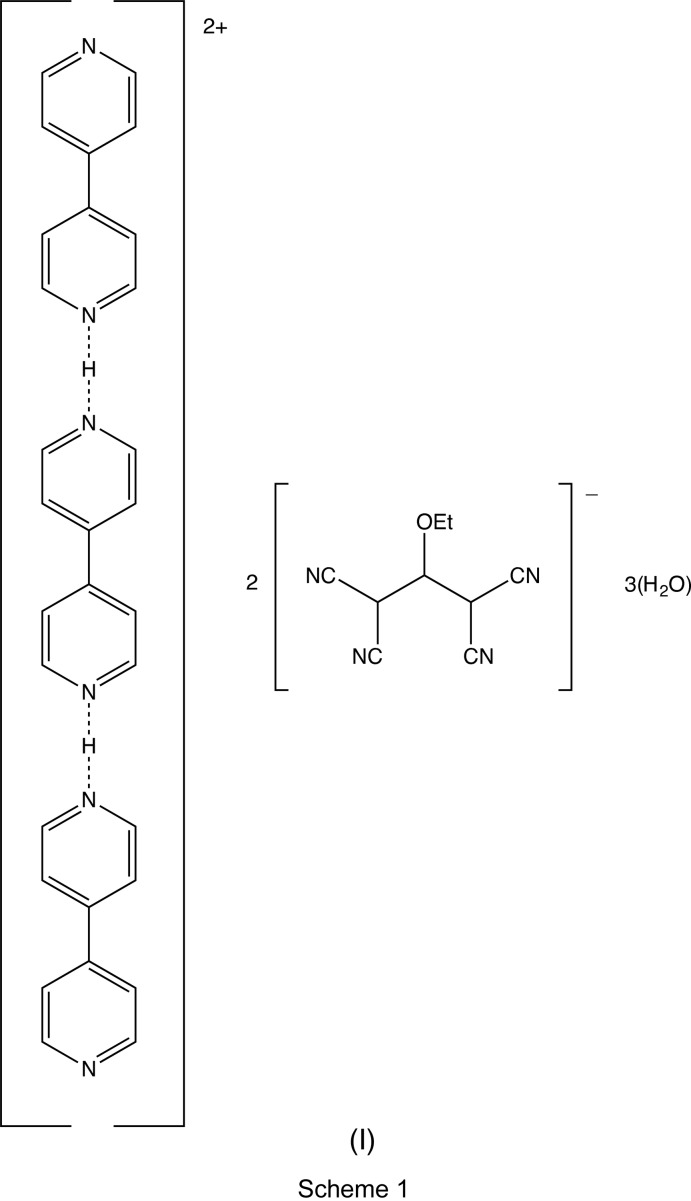


In the cation, the dihedral angle between the two symmetry-related rings of the central unit is 37.60 (4)°, the dihedral angle between the rings containing atoms N11 and N21 is 85.96 (5)° and that between the rings containing atoms N21 and N31 is 29.33 (3)° (*cf.* Fig. 1 ▸). In the anion, the corresponding pairs of bond distances and bond angles associated with the two C—C(CN)_2_ units containing the atoms C41 and C43 are very similar. In addition, the C—C distances in the C(CN)_2_ fragments are all short for their type [mean value (Allen *et al.*, 1987 ▸) 1.431 Å, lower quartile value 1.425 Å], while the C—N distances are all long for their type (mean value 1.136 Å, upper quartile value 1.142 Å). These observations indicate that there is considerable delocalization of the negative charge within the anion, not just over the central propenide fragment, resonance forms (a) and (b) (see Scheme 2), but also onto the N atoms of the four cyano substituents, forms (c)–(f). Despite this, the core skeleton of the anion is not planar, as the two C(CN)_2_ units are rotated in conrotatory fashion out of the plane of the propenide unit; this central C_3_ fragment makes dihedral angles of 10.39 (13) and 16.71 (18)°, respectively, with the C(CN)_2_ units containing atoms C41 and C43.
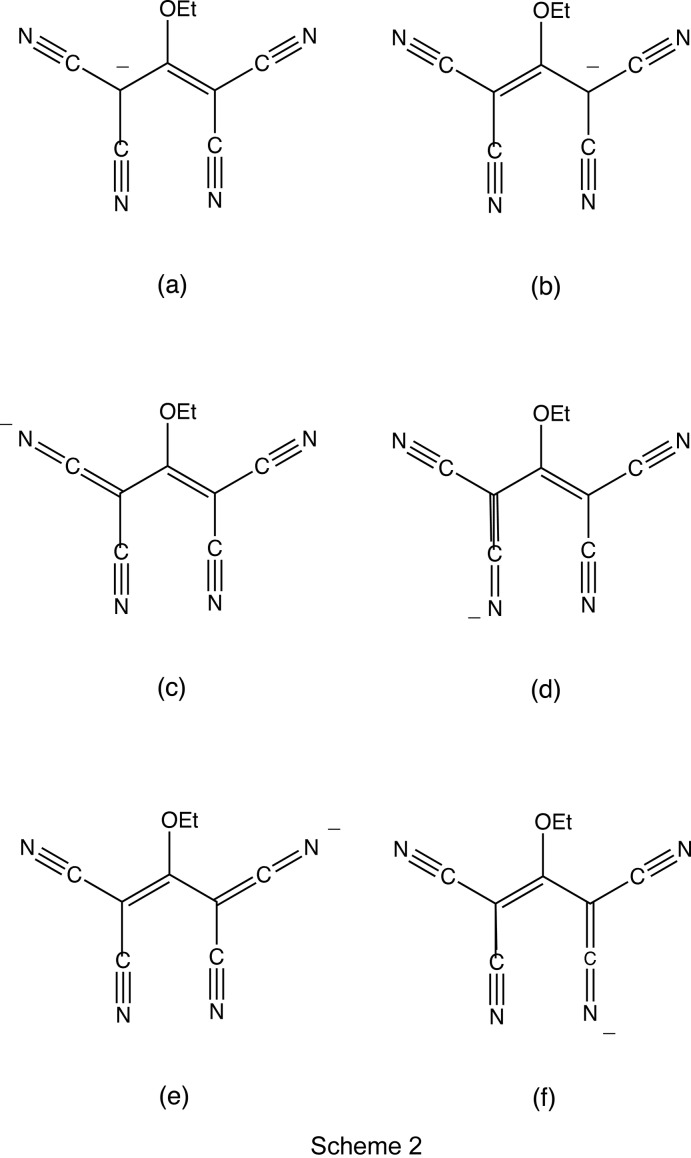


## Supra­molecular inter­actions   

The two independent 4,4′-bipy units are linked by disordered N—H⋯N hydrogen bonds, both of which are almost linear (Table 1 ▸). In addition, there are four C—H⋯N hydrogen bonds in the structure: two of these have donor atoms, C13 and C15, which are part of the 4,4′-bipy unit containing N11 and acceptors in the anion, one has an acceptor in the 4,4′-bipy unit containing N21 and N31, and the fourth involves only the anion. Of these four inter­actions, the first two can be regarded as charge-assisted hydrogen bonds (Gilli *et al.*, 1994 ▸) and it is inter­esting to note that the eth­oxy O atom in the anion plays no role in the supra­molecular assembly.

**Figure 2:**
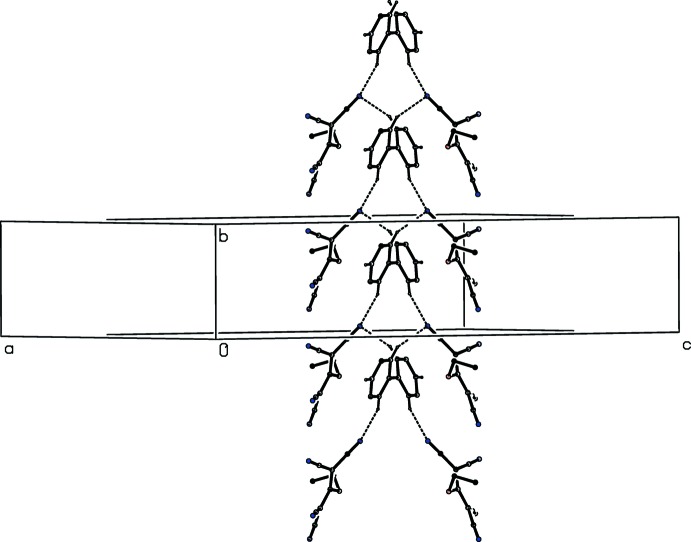
Part of the crystal structure of compound (I), showing the formation of a hydrogen-bonded ribbon of edge-fused 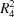
(14) rings along the [010] direction. For the sake of clarity, the 4,4′-bipy units containing atoms N21 and N31, the partial-occupancy water mol­ecules, and the H atoms not involved in the motif shown have been omitted.

**Figure 3:**
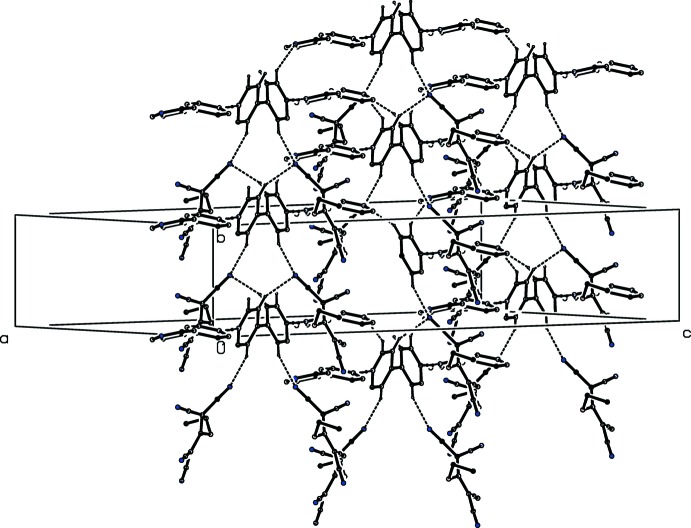
Part of the crystal structure of compound (I), showing the formation of a hydrogen-bonded sheet lying parallel to (10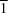). For the sake of clarity, the partial-occupancy water mol­ecules, and the H atoms not involved in the motif shown have been omitted.

**Figure 4:**
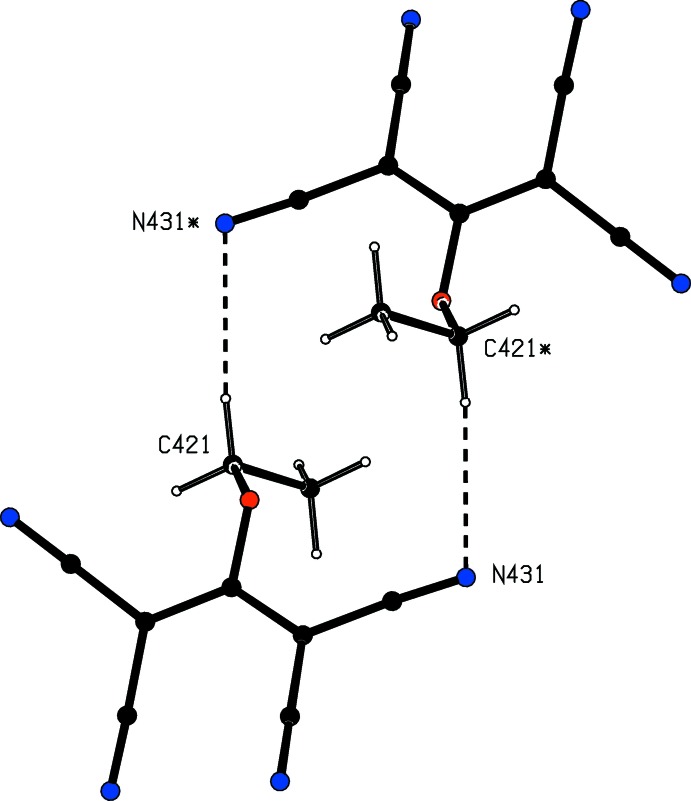
Part of the crystal structure of compound (I), showing the formation by pairs of anions of a hydrogen-bonded 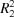
(14) ring. The atoms marked with an asterisk (*) are at the symmetry position (−*x*, −*y* + 1, −*z* + 1). For the sake of clarity, the unit-cell outline, the 4,4′-bipy units and the partial-occupancy water mol­ecules have all been omitted.

These six hydrogen bonds link the cations and anions into a three-dimensional network whose formation is readily analysed in terms of substructures (Ferguson *et al.*, 1998*a* ▸,*b* ▸; Gregson *et al.*, 2000 ▸) in zero, one and two dimensions. It is convenient to consider firstly the hydrogen bonds between cations and anions. The anions and the central 4,4′-bipy units containing atom N11 which are related by translation along the [010] direction are linked to form the one-dimensional substructure in the form of a ribbon of edge-fused 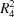
(14) loops (Fig. 2 ▸). Ribbons of this type, which are related by translation along [1
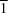
1], are linked by the 4,4′-bipy units containing atoms N21 and N31 to form the two-dimensional substructure, a sheet lying parallel to (10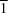) (Fig. 3 ▸). Adjacent sheets are linked by the zero-dimensional substructure which involves inversion-related pairs of anions forming a centrosymmetric motif characterized by an 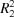
(14) ring (Fig. 4 ▸).

Three of the partially occupied water sites are linked by C—H⋯O hydrogen bonds (Table 1 ▸) within the selected asymmetric unit to one of the 4,4′-bipy components, while the fourth, O5*A*, lies 2.54 (3) Å from atom O6*A* at (−*x* + 1, *y* + 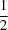, −*z* + 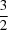), *i.e.* within the reference (10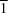) sheet and comfortably within O—H⋯O hydrogen-bonding range.

## Database survey   

The 1,1,3,3-tetra­cyano-2-eth­oxy­propenide unit, here conveniently denoted as *X* ^−^, has been reported in a number of structures. These include salts of organic cations, including [(2,2′-bipy)H]^+^·*X* ^−^, (II) (Setifi, Valkonen *et al.*, 2015 ▸), [(4,4′-bipy)H_2_]^2+^·2*X* ^−^, (III) (Setifi, Geiger *et al.*, 2015 ▸), and [(4,4′-bipy)Et_2_]^2+^·2*X* ^−^, (IV) (Setifi, Lehchili *et al.*, 2014 ▸); salts of mononuclear metal complexes in which the 1,1,3,3-tetra­cyano-2-eth­oxy­propenide unit is not ccordinated to the metal centre, including [(2,2′-bi-1*H*-imidazole)_2_Cu]^2+^·2*X* ^−^, (V) (Gaamoune *et al.*, 2010 ▸), [(1,10-phen)_3_Fe]^2−^·2*X* ^−^·0.5H_2_O, (VI) (Setifi, Setifi *et al.*, 2013 ▸), [(1,10-phen)_3_Fe]^2−^·2*X* ^−^·H_2_O, and (VII) (Setifi, Domasevitch *et al.*, 2013 ▸); and compounds where the 1,1,3,3-tetra­cyano-2-eth­oxy­propenide unit acts as a ligand including a binuclear Cu complex in which it acts both as a bridging ligand between two Cu^II^ centres and as a monodentate terminal ligand, thus [(2,2′-bipy)*X*Cu]_2_(μ-*X*)_2_, (VIII) (Addala *et al.*, 2015 ▸), and a two-dimensional coordination polymer [*X*(1,10-phen)ClCu]_*n*_, (IX) (Setifi, Setifi *et al.*, 2014 ▸).

Of these examples, compounds (II), (III) and (IV) are most closely related to compound (I) reported here. In the structure of compound (II), a combination of N—H⋯N and C—H⋯N hydrogen bonds links the ions into ribbons containing alternating 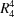
(18) and 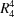
(26) rings; in (IV), where there are no N—H⋯N hydrogen bonds, the ions are linked into sheets by C—H⋯N hydrogen bonds, and in (III), an extensive series of N—H⋯N and C—H⋯N hydrogen bonds generates a three-dimensional network, so that the supra­molecular aggregation is one-, two- and three-dimensional in compounds (II), (IV) and (III), respectively.

## Synthesis and crystallization   

The salt K(tcnoet) was prepared according to a published method (Middleton *et al.*, 1958 ▸). The title compound was synthesized hydro­thermally under autogenous pressure from a mixture of iron(II) sulfate hepta­hydrate (56 mg, 0.2 mmol), 4,4′-bi­pyridine (32 mg, 0.2 mmol) and K(tcnoet) (90 mg, 0.4 mmol) in water–methanol (4:1 *v*/*v*, 20 ml). This mixture was sealed in a Teflon-lined autoclave and held at 423 K for 2 d, and then cooled to ambient temperature at a rate of 10 K h^−1^ (yield 25%). Pale-yellow blocks of the title compound suitable for single-crystal X-ray diffraction were selected directly from the synthesized product.

## Refinement   

Crystal data, data collection and structure refinement details are summarized in Table 2 ▸. The H atoms bonded to C or N atoms were all located in difference maps. The H atoms bonded to C atoms were subsequently treated as riding atoms in geometrically idealised positions, with C—H = 0.95 (pyrid­yl), 0.98 (CH_3_) or 0.99 Å (CH_2_), and with *U*_iso_(H) = *kU*_eq_(C) where *k* = 1.5 for the methyl group, which was permitted to rotate but not to tilt, and 1.2 for all other H atoms bonded to C atoms. The unique H atom bonded to N was disordered over two atomic sites, labeled H11 and H21, adjacent to atoms N11 and N21, respectively, and having unequal occupancies; for these two sites, the atomic coordinates were refined with *U*_iso_(H) = 1.2*U*_eq_(N), leading to the N—H distances shown in Table 1 ▸ and to refined site occupancies of 0.66 (4) and 0.36 (4) for H11 and H21, respectively. No H-atom sites associated with water atoms O5 and O6 could be located. Each of these water O atoms is disordered over two atomic sites: O5 is disordered over two sites, labelled O5*A* and O5*B*, which are separated by 0.963 (4) Å, while O6 is disordered over two sites, labelled O6*A* and O6*B*, which are separated by 0.627 (9) Å. Free refinement of the site occupancies of O5*A* and O5*B* gave values of 0.579 (7) and 0.512 (7), respectively; these values are not physically possible and both are over-estimates because of the lack of H atoms in the modelling of the water sites. Accordingly, the occupancies of O5*A* and O5*B* were constrained to sum to unity, giving values of 0.522 (6) and 0.478 (6). Free refinement of the site occupancies for O6*A* and O6*B* gave values of 0.36 (3) and 0.16 (3), and these values were subsequently restrained to sum to 0.500 (2), giving final values of 0.34 (3) and 0.16 (3). The final analysis of variance showed a large value, 4.522, of *K* = [mean(*F*_o_^2^)]/[mean(*F*_c_^2^)] for the group of 541 very weak reflections having *F*_c_/*F*_c_(max) in the range 0.000 < *F*_c_/*F*_c_(max) < 0.014.

## Supplementary Material

Crystal structure: contains datablock(s) global, I. DOI: 10.1107/S2056989016012160/hb7603sup1.cif

Structure factors: contains datablock(s) I. DOI: 10.1107/S2056989016012160/hb7603Isup2.hkl

CCDC reference: 1496221

Additional supporting information: crystallographic information; 3D view; checkCIF report

## Figures and Tables

**Table 1:** Hydrogen-bond geometry (Å, °)

*D*—H⋯*A*	*D*—H	H⋯*A*	*D*⋯*A*	*D*—H⋯*A*
N11—H11⋯N21	0.98 (4)	1.69 (4)	2.6655 (18)	175 (3)
N21—H21⋯N11	0.90 (7)	1.78 (7)	2.6655 (18)	172 (5)
C12—H12⋯N31^i^	0.95	2.57	3.4248 (19)	150
C13—H13⋯N411^ii^	0.95	2.56	3.434 (2)	154
C15—H15⋯N411	0.95	2.38	3.249 (2)	152
C25—H25⋯O5*B*	0.95	2.56	3.355 (4)	141
C35—H35⋯O6*A*	0.95	2.53	3.474 (13)	176
C35—H35⋯O6*B*	0.95	2.54	3.484 (16)	170
C421—H41*A*⋯N431^iii^	0.99	2.61	3.589 (2)	172

Symmetry codes: (i) 
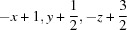
; (ii) 
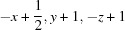
; (iii) 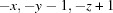.

**Table 2:** Experimental details

Crystal data	
Chemical formula	C_30_H_26_N_6_^2+^·2C_9_H_5_N_4_O^−^·3H_2_O
*M*_r_	894.95
Crystal system, space group	Monoclinic, *I*2/*a*
Temperature (K)	123
*a*, *b*, *c* (Å)	18.1861 (2), 7.1187 (1), 35.7070 (4)
β (°)	100.448 (1)
*V* (Å^3^)	4546.03 (10)
*Z*	4
Radiation type	Mo *K*α
μ (mm^−1^)	0.09
Crystal size (mm)	0.45 × 0.38 × 0.31

Data collection	
Diffractometer	Bruker–Nonius Kappa CCD with APEXII detector
Absorption correction	Multi-scan (*SADABS*; Sheldrick, 2003 ▸)
*T*_min_, *T*_max_	0.907, 0.973
No. of measured, independent and observed [*I* > 2σ(*I*)] reflections	35680, 5197, 4559
*R*_int_	0.039
(sin θ/λ)_max_ (Å^−1^)	0.650

Refinement	
*R*[*F* ^2^ > 2σ(*F* ^2^)], *wR*(*F* ^2^), *S*	0.049, 0.115, 1.09
No. of reflections	5197
No. of parameters	335
No. of restraints	1
H-atom treatment	H atoms treated by a mixture of independent and constrained refinement
Δρ_max_, Δρ_min_ (e Å^−3^)	0.32, −0.23

Computer programs: *COLLECT* (Bruker, 2008 ▸), *DENZO-SMN* (Otwinowski & Minor, 1997 ▸), *SHELXS97* (Sheldrick, 2008 ▸), *SHELXL2014* (Sheldrick, 2015 ▸) and *PLATON* (Spek, 2009 ▸).

## supplementary crystallographic information

### Crystal data

**Table:**

C_30_H_26_N_6_^2+^·2C_9_H_5_N_4_O^−^·3H_2_O	*F*(000) = 1872
*M**_r_* = 894.95	*D*_x_ = 1.299 Mg m^−^^3^
Monoclinic, *I*2/*a*	Mo *K*α radiation, λ = 0.71073 Å
*a* = 18.1861 (2) Å	Cell parameters from 5197 reflections
*b* = 7.1187 (1) Å	θ = 2.3–27.5°
*c* = 35.7070 (4) Å	µ = 0.09 mm^−^^1^
β = 100.448 (1)°	*T* = 123 K
*V* = 4546.03 (10) Å^3^	Block, pale yellow
*Z* = 4	0.45 × 0.38 × 0.31 mm

### Data collection

**Table:**

Bruker–Nonius Kappa CCD with APEXII detector diffractometer	5197 independent reflections
Radiation source: fine focus sealed tube	4559 reflections with *I* > 2σ(*I*)
Graphite monochromator	*R*_int_ = 0.039
φ and ω scans	θ_max_ = 27.5°, θ_min_ = 2.3°
Absorption correction: multi-scan (SADABS; Sheldrick, 2003)	*h* = −23→23
*T*_min_ = 0.907, *T*_max_ = 0.973	*k* = −8→9
35680 measured reflections	*l* = −42→46

### Refinement

**Table:**

Refinement on *F*^2^	1 restraint
Least-squares matrix: full	Hydrogen site location: mixed
*R*[*F*^2^ > 2σ(*F*^2^)] = 0.049	H atoms treated by a mixture of independent and constrained refinement
*wR*(*F*^2^) = 0.115	*w* = 1/[σ^2^(*F*_o_^2^) + (0.0362*P*)^2^ + 5.2868*P*] where *P* = (*F*_o_^2^ + 2*F*_c_^2^)/3
*S* = 1.09	(Δ/σ)_max_ = 0.001
5197 reflections	Δρ_max_ = 0.32 e Å^−^^3^
335 parameters	Δρ_min_ = −0.23 e Å^−^^3^

### Special details

**Table:**

Geometry. All esds (except the esd in the dihedral angle between two l.s. planes) are estimated using the full covariance matrix. The cell esds are taken into account individually in the estimation of esds in distances, angles and torsion angles; correlations between esds in cell parameters are only used when they are defined by crystal symmetry. An approximate (isotropic) treatment of cell esds is used for estimating esds involving l.s. planes.

### Fractional atomic coordinates and isotropic or equivalent isotropic displacement parameters (Å^2^)

**Table:**

	*x*	*y*	*z*	*U*_iso_*/*U*_eq_	Occ. (<1)
N11	0.34943 (7)	0.62579 (18)	0.59359 (3)	0.0268 (3)	
H11	0.3778 (17)	0.621 (4)	0.6196 (11)	0.032*	0.66 (4)
C12	0.34888 (8)	0.7810 (2)	0.57234 (4)	0.0260 (3)	
H12	0.3753	0.8893	0.5830	0.031*	
C13	0.31054 (7)	0.7862 (2)	0.53523 (4)	0.0234 (3)	
H13	0.3109	0.8967	0.5204	0.028*	
C14	0.27134 (7)	0.62756 (19)	0.51984 (3)	0.0192 (3)	
C15	0.27272 (8)	0.4676 (2)	0.54248 (4)	0.0244 (3)	
H15	0.2466	0.3574	0.5327	0.029*	
C16	0.31261 (8)	0.4715 (2)	0.57924 (4)	0.0276 (3)	
H16	0.3140	0.3625	0.5947	0.033*	
N21	0.42091 (7)	0.62202 (17)	0.66569 (4)	0.0269 (3)	
H21	0.400 (3)	0.616 (8)	0.641 (2)	0.032*	0.34 (4)
C22	0.49341 (8)	0.5785 (2)	0.67630 (4)	0.0276 (3)	
H22	0.5217	0.5488	0.6572	0.033*	
C23	0.52856 (8)	0.5754 (2)	0.71399 (4)	0.0256 (3)	
H23	0.5802	0.5455	0.7206	0.031*	
C24	0.48726 (8)	0.61675 (19)	0.74231 (4)	0.0219 (3)	
C25	0.41210 (8)	0.6605 (2)	0.73098 (4)	0.0258 (3)	
H25	0.3821	0.6887	0.7494	0.031*	
C26	0.38118 (8)	0.6626 (2)	0.69263 (4)	0.0277 (3)	
H26	0.3298	0.6940	0.6852	0.033*	
N31	0.58874 (8)	0.61811 (19)	0.86102 (3)	0.0331 (3)	
C32	0.51622 (9)	0.5772 (2)	0.84948 (4)	0.0327 (3)	
H32	0.4872	0.5488	0.8683	0.039*	
C33	0.48101 (9)	0.5739 (2)	0.81152 (4)	0.0281 (3)	
H33	0.4294	0.5438	0.8049	0.034*	
C34	0.52250 (8)	0.61556 (19)	0.78334 (4)	0.0227 (3)	
C35	0.59782 (8)	0.6576 (2)	0.79502 (4)	0.0270 (3)	
H35	0.6283	0.6867	0.7768	0.032*	
C36	0.62787 (9)	0.6565 (2)	0.83360 (4)	0.0313 (3)	
H36	0.6795	0.6848	0.8410	0.038*	
C41	0.15157 (8)	−0.1715 (2)	0.58797 (4)	0.0272 (3)	
C42	0.11579 (7)	−0.3411 (2)	0.57565 (4)	0.0234 (3)	
C43	0.11642 (8)	−0.5041 (2)	0.59765 (4)	0.0249 (3)	
C411	0.16626 (8)	−0.0335 (2)	0.56164 (4)	0.0297 (3)	
N411	0.17940 (8)	0.0773 (2)	0.54035 (4)	0.0382 (3)	
C412	0.17576 (9)	−0.1283 (2)	0.62709 (5)	0.0347 (4)	
N412	0.19482 (10)	−0.0875 (3)	0.65855 (5)	0.0528 (4)	
C431	0.06545 (8)	−0.6532 (2)	0.58565 (4)	0.0262 (3)	
N431	0.02403 (7)	−0.77540 (19)	0.57765 (4)	0.0333 (3)	
C432	0.16949 (9)	−0.5343 (2)	0.63136 (4)	0.0321 (3)	
N432	0.21320 (9)	−0.5619 (3)	0.65832 (4)	0.0496 (4)	
O421	0.08005 (6)	−0.35955 (15)	0.53932 (3)	0.0283 (2)	
C421	0.02174 (9)	−0.2228 (2)	0.52443 (4)	0.0330 (3)	
H41A	0.0143	−0.2200	0.4963	0.040*	
H41B	0.0372	−0.0959	0.5341	0.040*	
C422	−0.04964 (9)	−0.2764 (3)	0.53674 (5)	0.0427 (4)	
H42A	−0.0886	−0.1850	0.5268	0.064*	
H42B	−0.0650	−0.4016	0.5269	0.064*	
H42C	−0.0421	−0.2778	0.5646	0.064*	
O5A	0.2512 (2)	0.9317 (7)	0.73743 (12)	0.0915 (17)	0.522 (6)
O5B	0.28182 (16)	0.9204 (5)	0.76234 (11)	0.0533 (13)	0.478 (6)
O6A	0.7139 (8)	0.743 (4)	0.7296 (2)	0.055 (4)	0.34 (3)
O6B	0.7007 (9)	0.824 (4)	0.7286 (3)	0.031 (4)	0.16 (3)

### Atomic displacement parameters (Å^2^)

**Table:**

	*U*^11^	*U*^22^	*U*^33^	*U*^12^	*U*^13^	*U*^23^
N11	0.0253 (6)	0.0364 (7)	0.0166 (5)	0.0030 (5)	−0.0018 (5)	0.0009 (5)
C12	0.0241 (7)	0.0309 (8)	0.0210 (6)	−0.0013 (6)	−0.0013 (5)	−0.0028 (6)
C13	0.0248 (6)	0.0246 (7)	0.0199 (6)	−0.0014 (5)	0.0015 (5)	0.0007 (5)
C14	0.0165 (6)	0.0233 (7)	0.0169 (6)	0.0020 (5)	0.0011 (5)	0.0004 (5)
C15	0.0244 (6)	0.0254 (7)	0.0224 (7)	−0.0016 (6)	0.0013 (5)	0.0016 (5)
C16	0.0295 (7)	0.0306 (8)	0.0217 (7)	0.0026 (6)	0.0020 (5)	0.0063 (6)
N21	0.0325 (6)	0.0262 (6)	0.0188 (6)	−0.0020 (5)	−0.0036 (5)	0.0016 (5)
C22	0.0322 (7)	0.0283 (7)	0.0216 (7)	−0.0004 (6)	0.0034 (6)	0.0011 (6)
C23	0.0259 (7)	0.0255 (7)	0.0235 (7)	0.0015 (6)	−0.0002 (5)	0.0007 (5)
C24	0.0269 (7)	0.0174 (6)	0.0197 (6)	−0.0014 (5)	0.0001 (5)	0.0006 (5)
C25	0.0270 (7)	0.0265 (7)	0.0228 (7)	−0.0006 (6)	0.0016 (5)	−0.0024 (5)
C26	0.0260 (7)	0.0280 (8)	0.0259 (7)	−0.0003 (6)	−0.0035 (5)	−0.0002 (6)
N31	0.0437 (8)	0.0285 (7)	0.0227 (6)	0.0020 (6)	−0.0053 (5)	0.0004 (5)
C32	0.0466 (9)	0.0287 (8)	0.0216 (7)	−0.0022 (7)	0.0029 (6)	0.0032 (6)
C33	0.0332 (7)	0.0253 (7)	0.0239 (7)	−0.0026 (6)	0.0003 (6)	0.0014 (6)
C34	0.0295 (7)	0.0174 (6)	0.0193 (6)	0.0035 (5)	−0.0010 (5)	−0.0005 (5)
C35	0.0292 (7)	0.0249 (7)	0.0250 (7)	0.0031 (6)	0.0001 (6)	−0.0028 (5)
C36	0.0324 (8)	0.0294 (8)	0.0280 (7)	0.0037 (6)	−0.0054 (6)	−0.0041 (6)
C41	0.0283 (7)	0.0256 (7)	0.0286 (7)	−0.0013 (6)	0.0074 (6)	−0.0023 (6)
C42	0.0226 (6)	0.0266 (7)	0.0217 (6)	0.0015 (5)	0.0060 (5)	−0.0009 (5)
C43	0.0246 (7)	0.0266 (7)	0.0233 (7)	−0.0006 (6)	0.0038 (5)	0.0012 (5)
C411	0.0284 (7)	0.0245 (7)	0.0379 (8)	−0.0001 (6)	0.0108 (6)	−0.0039 (6)
N411	0.0419 (8)	0.0267 (7)	0.0495 (8)	−0.0033 (6)	0.0181 (7)	0.0013 (6)
C412	0.0343 (8)	0.0332 (9)	0.0375 (9)	−0.0080 (7)	0.0084 (7)	−0.0069 (7)
N412	0.0583 (10)	0.0593 (11)	0.0399 (9)	−0.0190 (8)	0.0065 (7)	−0.0157 (8)
C431	0.0278 (7)	0.0265 (7)	0.0250 (7)	0.0036 (6)	0.0068 (5)	0.0037 (6)
N431	0.0345 (7)	0.0284 (7)	0.0366 (7)	−0.0030 (6)	0.0053 (6)	0.0006 (6)
C432	0.0305 (8)	0.0326 (8)	0.0325 (8)	−0.0031 (6)	0.0036 (6)	0.0064 (6)
N432	0.0429 (8)	0.0560 (10)	0.0432 (9)	−0.0030 (7)	−0.0096 (7)	0.0147 (7)
O421	0.0348 (5)	0.0277 (5)	0.0211 (5)	0.0027 (4)	0.0019 (4)	−0.0006 (4)
C421	0.0368 (8)	0.0334 (8)	0.0263 (7)	0.0047 (7)	−0.0011 (6)	0.0061 (6)
C422	0.0353 (9)	0.0423 (10)	0.0489 (10)	0.0042 (8)	0.0031 (7)	−0.0003 (8)
O5A	0.059 (2)	0.166 (4)	0.054 (3)	0.015 (2)	0.021 (2)	0.037 (2)
O5B	0.0324 (15)	0.082 (2)	0.043 (2)	0.0148 (14)	0.0002 (14)	−0.0208 (16)
O6A	0.058 (4)	0.072 (11)	0.038 (2)	−0.021 (6)	0.015 (2)	−0.009 (4)
O6B	0.047 (5)	0.023 (9)	0.027 (4)	0.002 (5)	0.016 (3)	0.000 (4)

### Geometric parameters (Å, º)

**Table:**

N11—C16	1.339 (2)	C33—C34	1.395 (2)
N11—C12	1.3393 (19)	C33—H33	0.9500
N11—H11	0.98 (4)	C34—C35	1.390 (2)
C12—C13	1.3813 (19)	C35—C36	1.387 (2)
C12—H12	0.9500	C35—H35	0.9500
C13—C14	1.3938 (19)	C36—H36	0.9500
C13—H13	0.9500	C41—C42	1.404 (2)
C14—C15	1.3943 (19)	C41—C411	1.418 (2)
C14—C14^i^	1.487 (2)	C41—C412	1.420 (2)
C15—C16	1.3802 (19)	C42—O421	1.3480 (16)
C15—H15	0.9500	C411—N411	1.151 (2)
C16—H16	0.9500	C412—N412	1.151 (2)
N21—C26	1.335 (2)	C42—C43	1.400 (2)
N21—C22	1.3405 (19)	C43—C431	1.423 (2)
N21—H21	0.90 (8)	C43—C432	1.416 (2)
C22—C23	1.3813 (19)	C431—N431	1.152 (2)
C22—H22	0.9500	C432—N432	1.149 (2)
C23—C24	1.396 (2)	O421—C421	1.4662 (18)
C23—H23	0.9500	C421—C422	1.493 (2)
C24—C25	1.3887 (19)	C421—H41A	0.9900
C24—C34	1.4885 (18)	C421—H41B	0.9900
C25—C26	1.3826 (19)	C422—H42A	0.9800
C25—H25	0.9500	C422—H42B	0.9800
C26—H26	0.9500	C422—H42C	0.9800
N31—C36	1.339 (2)	O5A—O5B	0.963 (4)
N31—C32	1.340 (2)	O6A—O6B	0.627 (9)
C32—C33	1.390 (2)	O6A—O6A^ii^	1.78 (2)
C32—H32	0.9500		
			
C16—N11—C12	120.54 (12)	C32—C33—C34	119.08 (14)
C16—N11—H11	118.5 (16)	C32—C33—H33	120.5
C12—N11—H11	121.0 (17)	C34—C33—H33	120.5
N11—C12—C13	121.05 (14)	C35—C34—C33	117.49 (13)
N11—C12—H12	119.5	C35—C34—C24	121.27 (13)
C13—C12—H12	119.5	C33—C34—C24	121.24 (13)
C12—C13—C14	119.33 (13)	C36—C35—C34	119.10 (14)
C12—C13—H13	120.3	C36—C35—H35	120.5
C14—C13—H13	120.3	C34—C35—H35	120.5
C13—C14—C15	118.63 (11)	N31—C36—C35	124.16 (14)
C13—C14—C14^i^	121.13 (8)	N31—C36—H36	117.9
C15—C14—C14^i^	120.25 (9)	C35—C36—H36	117.9
C16—C15—C14	119.06 (13)	C42—C41—C411	121.34 (13)
C16—C15—H15	120.5	C42—C41—C412	122.53 (14)
C14—C15—H15	120.5	C411—C41—C412	116.12 (14)
N11—C16—C15	121.39 (13)	O421—C42—C43	114.36 (12)
N11—C16—H16	119.3	O421—C42—C41	120.05 (13)
C15—C16—H16	119.3	C41—C42—C43	125.51 (13)
C26—N21—C22	118.63 (12)	C42—C43—C431	120.70 (13)
C26—N21—H21	123 (3)	C42—C43—C432	122.51 (13)
C22—N21—H21	119 (3)	C431—C43—C432	116.70 (13)
N21—C22—C23	122.47 (14)	N411—C411—C41	178.83 (16)
N21—C22—H22	118.8	N412—C412—C41	177.9 (2)
C23—C22—H22	118.8	N431—C431—C43	176.87 (15)
C22—C23—C24	119.19 (13)	N432—C432—C43	178.5 (2)
C22—C23—H23	120.4	C42—O421—C421	118.36 (11)
C24—C23—H23	120.4	O421—C421—C422	109.51 (13)
C25—C24—C23	117.80 (12)	O421—C421—H41A	109.8
C25—C24—C34	120.90 (12)	C422—C421—H41A	109.8
C23—C24—C34	121.30 (12)	O421—C421—H41B	109.8
C26—C25—C24	119.55 (13)	C422—C421—H41B	109.8
C26—C25—H25	120.2	H41A—C421—H41B	108.2
C24—C25—H25	120.2	C421—C422—H42A	109.5
N21—C26—C25	122.35 (13)	C421—C422—H42B	109.5
N21—C26—H26	118.8	H42A—C422—H42B	109.5
C25—C26—H26	118.8	C421—C422—H42C	109.5
C36—N31—C32	116.31 (13)	H42A—C422—H42C	109.5
N31—C32—C33	123.86 (15)	H42B—C422—H42C	109.5
N31—C32—H32	118.1	O6B—O6A—O6A^ii^	102 (2)
C33—C32—H32	118.1		
			
C16—N11—C12—C13	0.0 (2)	C25—C24—C34—C35	−150.27 (14)
N11—C12—C13—C14	0.5 (2)	C23—C24—C34—C35	29.3 (2)
C12—C13—C14—C15	−0.6 (2)	C25—C24—C34—C33	29.1 (2)
C12—C13—C14—C14^i^	179.35 (14)	C23—C24—C34—C33	−151.33 (14)
C13—C14—C15—C16	0.1 (2)	C33—C34—C35—C36	−0.1 (2)
C14^i^—C14—C15—C16	−179.83 (14)	C24—C34—C35—C36	179.35 (13)
C12—N11—C16—C15	−0.5 (2)	C32—N31—C36—C35	0.6 (2)
C14—C15—C16—N11	0.4 (2)	C34—C35—C36—N31	−0.4 (2)
C26—N21—C22—C23	−0.4 (2)	C411—C41—C42—O421	−14.7 (2)
N21—C22—C23—C24	0.8 (2)	C412—C41—C42—O421	165.94 (14)
C22—C23—C24—C25	−0.3 (2)	C411—C41—C42—C43	161.79 (14)
C22—C23—C24—C34	−179.91 (13)	C412—C41—C42—C43	−17.6 (2)
C23—C24—C25—C26	−0.3 (2)	O421—C42—C43—C432	158.96 (13)
C34—C24—C25—C26	179.24 (13)	C41—C42—C43—C432	−17.7 (2)
C22—N21—C26—C25	−0.3 (2)	O421—C42—C43—C431	−17.47 (19)
C24—C25—C26—N21	0.7 (2)	C41—C42—C43—C431	165.87 (14)
C36—N31—C32—C33	−0.3 (2)	C43—C42—O421—C421	127.63 (14)
N31—C32—C33—C34	−0.1 (2)	C41—C42—O421—C421	−55.52 (18)
C32—C33—C34—C35	0.3 (2)	C42—O421—C421—C422	−81.41 (17)
C32—C33—C34—C24	−179.10 (13)		

Symmetry codes: (i) −*x*+1/2, *y*, −*z*+1; (ii) −*x*+3/2, −*y*+3/2, −*z*+3/2.

### Hydrogen-bond geometry (Å, º)

**Table:**

*D*—H···*A*	*D*—H	H···*A*	*D*···*A*	*D*—H···*A*
N11—H11···N21	0.98 (4)	1.69 (4)	2.6655 (18)	175 (3)
N21—H21···N11	0.90 (7)	1.78 (7)	2.6655 (18)	172 (5)
C12—H12···N31^iii^	0.95	2.57	3.4248 (19)	150
C13—H13···N411^iv^	0.95	2.56	3.434 (2)	154
C15—H15···N411	0.95	2.38	3.249 (2)	152
C25—H25···O5*B*	0.95	2.56	3.355 (4)	141
C35—H35···O6*A*	0.95	2.53	3.474 (13)	176
C35—H35···O6*B*	0.95	2.54	3.484 (16)	170
C421—H41*A*···N431^v^	0.99	2.61	3.589 (2)	172

Symmetry codes: (iii) −*x*+1, *y*+1/2, −*z*+3/2; (iv) −*x*+1/2, *y*+1, −*z*+1; (v) −*x*, −*y*−1, −*z*+1.
